# Diagnosis pitfall of interstitial pregnancy: a case report of a term pregnancy with abnormal placentation

**DOI:** 10.1186/s12884-021-04153-1

**Published:** 2021-10-18

**Authors:** Fatemeh Sadat Najib, Homeira Vafaei, Amin Abolhasan Foroughi, Niloofar Namazi

**Affiliations:** 1 Infertility Research Center, Shiraz University of Medical Sciences, Shiraz, Iran; 2 Department of Obstetrics and Gynecology, School of Medicine, Shiraz University of Medical Sciences, Shiraz, Iran; 3 Maternal-fetal Medicine Research Center, Shiraz University of Medical Sciences, Shiraz, Iran; 4 Medical Imaging Research Center, Shiraz University of Medical Sciences, Shiraz, Iran; 5 Department of Radiology, School of Medicine, Shiraz University of Medical Sciences, Shiraz, Iran

**Keywords:** Interstitial pregnancy, Cancer, Case report, Placenta increta, Term

## Abstract

**Background:**

Interstitial Pregnancy (IP) is a lethal condition due to high risk of sudden onset massive hemorrhage. Such conditions are managed as soon as diagnosed almost in the first trimester. There are a few case reports of IP conditions terminated after the second trimester. Herein, we introduce a term interstitial pregnancy complicated by abnormal placentation.

**Case presentation:**

In this case report, we introduce a 32-year-old lady, primigravida, with an undiagnosed IP that was in her 38 weeks of gestational with placenta increta. She developed with perforated IP presenting with acute abdomen and internal bleeding at 26 weeks of gestational age. However, with a misdiagnosis impression, she got stable in operation room. Then, the pregnancy continued till 36 weeks of gestational age that was misdiagnosed with cervical cancer in prenatal work-ups. Finally, during elective cesarean section at 38 weeks, an IP with placenta increta (placenta evading from the serosa to the myometrium of the uterus) was observed. The baby was healthy with no obvious anomaly or morbidity.

**Conclusions:**

Physicians should be aware to detect IP in all trimesters and pay attention to the coexisting complications such as placenta accreta to manage them more accurately.

## Background

Interstitial pregnancy (IP) is defined as a kind of ectopic pregnancy in which the pregnancy product is implanted in the intramural fragment of the tube. It is estimated that it accounts for less than 3% of ectopic pregnancies [1, 2]. Although it may remain asymptomatic in the first 14–16 weeks of pregnancy [3], it is mostly ruptured after the first trimester that leads to massive hemorrhage due to high vascularity [4]. Considering to this fact, there are few reports of full-term interstitial pregnancies [4, 5]. Also, there is likelihood of the presence of abnormal placentation in this category of ectopic pregnancy that make this condition more complicated[2, 4, 5]. The rarity of this case report is delivery a full term misdiagnosed interstitial pregnancy complicated by placenta increta. In this case, the ruptured IP was stabilized during pregnancy and her pregnancy continued as a normal intra-uterine pregnancy. Also, high vascularity of abnormal placentation (that was entered from serosa to  myometrium) in MRI was another diagnosis pitfall that was labeled as cervical cancer. The correct diagnosis was established during cesarean section.

## Case presentation

A 32-year-old Iranian woman, primigravida, with 38 weeks of gestational age was referred to our center due to her high risk obstetrical course. No significant point was detected in her thorough physical examination. She had no history of infertility or any significant medical disease or surgery before pregnancy. Her first trimester of pregnancy had normal course with no pathologic finding in sonography or lab data. When she was at 26 weeks of gestational age, she developed with acute abdomen and vomiting and was transferred to the operation room by an obstetrician in her local area. Her operation notes revealed a 4.5 cm degenerated posterior wall uterine myoma with active bleeding and about 3 liters of intra-abdominal blood loss. Patient hemostasis by suturing and blood transfusion was performed.

**Fig. 1:**
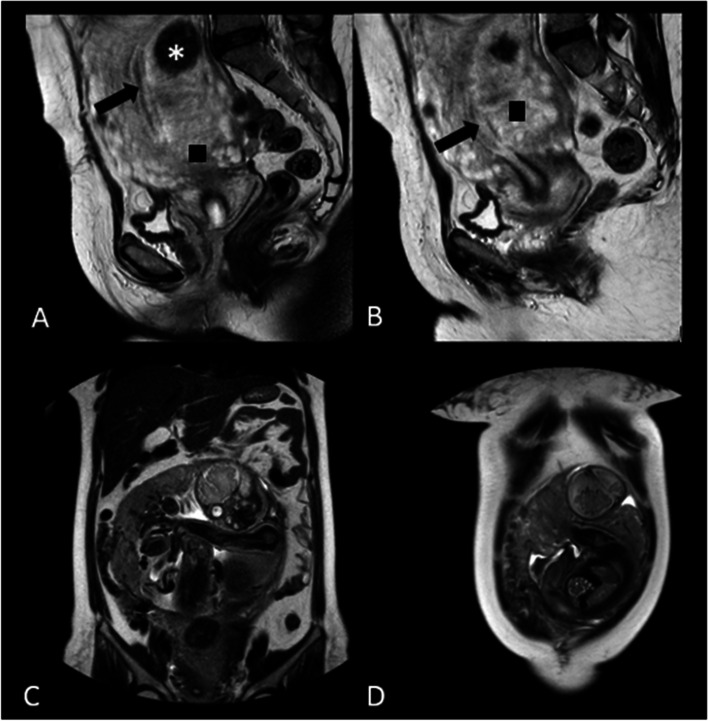
Interstitial pregnancy. Initial interpretation of magnetic resonance images revealed heterogenous hypersignal structure within the lower segment of the uterus that seemed to invade the cervical stroma () which erroneously was diagnosed as lower uterine/cervical mass; however, surgical findings confirmed that this was abnormally adherent invasive placenta of interstitial pregnancy. Retrospective re-evaluations of MRI were as follows: Sagittal (**A**, **B**) T2-weighted HASTE MR images showing intramural myoma (*) within the posterior wall of the uterus. Note that the endometrial cavity is completely empty (arrow). Coronal (**C**, **D**) T2-weighted HASTE MR images (obtained more cranial than A, B at the level of abdominal cavity) show eccentrically located fetus with overlying thin myometrium. Relationship of pregnancy and utero-tubal junction or the interstitial portion of fallopian tubes was imprecise as a result of the fetus size. The patient underwent successful termination via C-section and the finding at surgery confirmed the interstitial pregnancy

At gestational age of 36 weeks and 4 days, she was referred to our high-risk perinatology clinic affiliated to Shiraz University of Medical Sciences which is a referral center of perinatology in the south of Iran due to suspicion to cervical malignancy in a local sonography. In perinatology ward sonography, there was a single living fetus with breech presentation. The biometry revealed a biparietal diameter of 97th centile, abdominal circumference of 23rd centile, and estimated fetal weight of 2851, which is the 41st centile for 36 weeks and 4 days of gestational age. Reassuring pattern of color Doppler and normal amniotic fluid index (14 cm) were reported. There were multiple hypo-echoic areas in the placenta as well as a heterogeneous mass measuring 77*88*66 mm in the lower part of the uterus. For further evaluation, pelvic MRI was performed, reporting enlarged uterus with circumferential mass-like thickening in the lower segment of the uterus with vascularity, leading to differential diagnosis of cervical cancer (Fig. 1). In addition to multiple non-significant uterine myomas, a well-defined iso-signal in T1 and heterogeneous hypo-signal in T2 structure, measuring 29*39 mm, was diagnosed in the lower segment.

**Fig. 2:**
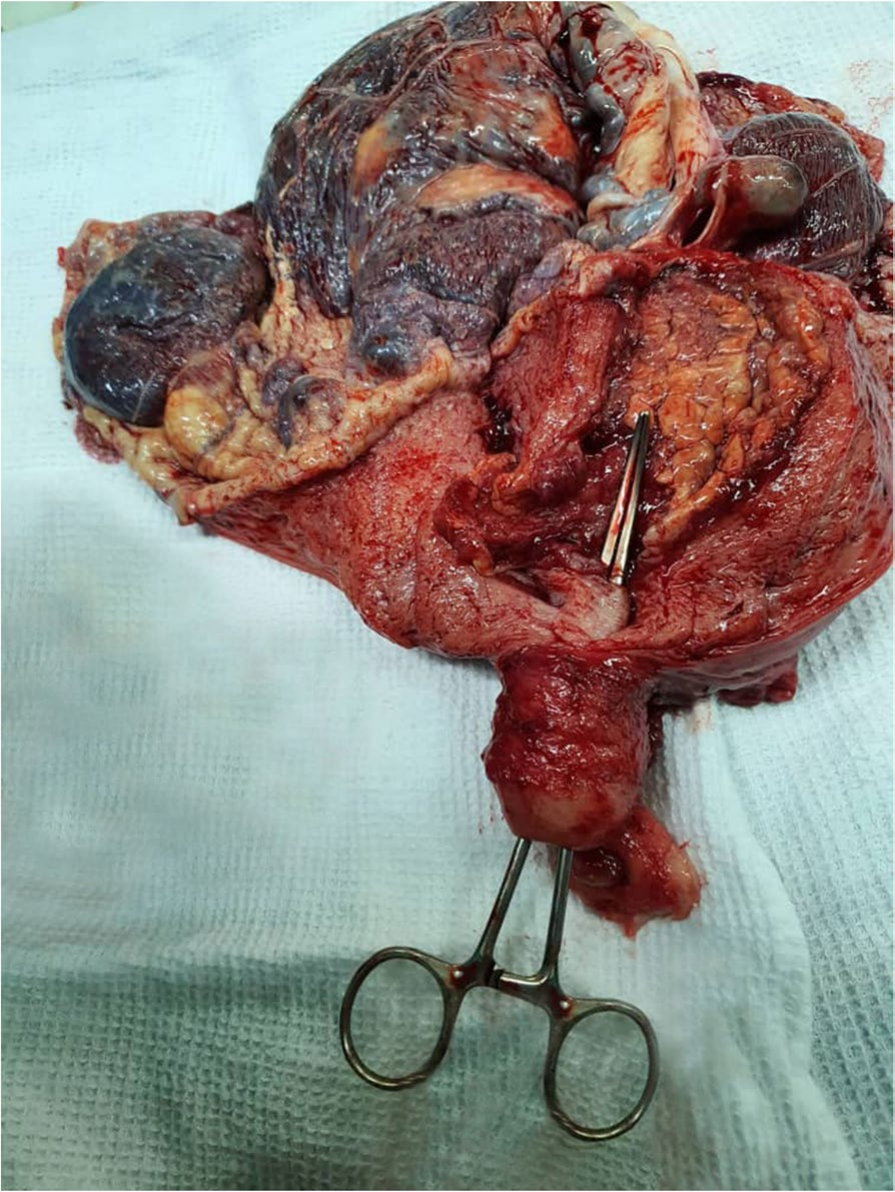
Graph of the uterus and placenta. The clamp shows the empty uterus. Note the reverse implantation of placenta increta invading from the serosa of the uterus to the myometrium

**Fig. 3:**
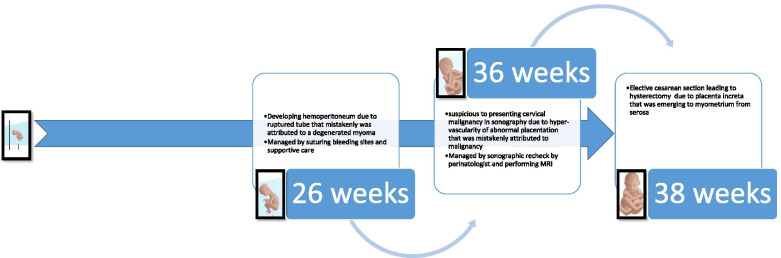
Pregnancy Timeline presenting events during pregnancy

At 38 weeks of gestational age, the patient underwent cesarean section. The rare finding of term interstitial pregnancy was found with placenta increta formation that was evading to the myometrium from the serosa (reversely from outside to the inside of the uterus) that was confirmed by pathology study (Fig. 2). A boy weighing 2840 g with an APGAR score of 8 to 9 in the first and 5th min of birth was delivered. Hysterectomy was done. Nothing significant in the post-operation course was noted. These events are summarized in Fig. 3, using a timeline.

## Discussion and conclusions

This case report shows a term interstitial pregnancy with abnormal implantation of the placenta. In a study published in 2020 [2], the authors report 6 term interstitial pregnancies, one of which developed with preeclampsia and the others had no specific event in their in pregnancy course. Also, there are reports of abnormal placentation in this condition. To explain more, one premature third trimester pregnancy presented with myometrial infiltrating placenta [6]. Kakigano et al. presented another case report of asymmetrical appearance of the uterus with final diagnosis of interstitial pregnancy with placenta accreta [4]. Also, there is another report of an interstitial pregnancy with placenta percreta, which led to the birth of a viable fetus [5]. Contrary to the literature, in our case, no pathology was found in her previous scans, and she was managed as a normal intra-uterine pregnancy. Also, when this IP was ruptured and the patient developed with acute abdomen and internal bleeding, the patient underwent surgery and stabilized in the aspect of the blood loss with a misdiagnosed impression. After termination, a term IP with placenta increta, emerging from the serosa to the myometrium, was diagnosed that was the reason of high vascularity around the cervix in her MRI leading to misdiagnosis of cervical cancer (Fig. 3).

Interstitial pregnancy management of early pregnancy is by either medical therapy or surgery, each appropriate for the selected group [7]. To add some details, some factors like the size of the gestational sac and the time of diagnosis can affect the success rate of medical therapy [7, 8], while the surgical method is introduced as a risk factor for cesarean section [9]. The selected method of surgery has variations based on the duration and outcome of the surgery as laparoscopic cornuostomy is introduced as the method of choice [8]. Although this method increases the risk of developing rupture uterus in the future pregnancy [10], laparoscopic approach is introduced to be the first line of therapy in a recent meta-analysis with the benefit of average bleeding of 168 cc that depends on the gravidity and duration of the amenorrhea that the patient has [11]. Focusing on the operative management of IP, especially the retained tissues, the benefit of hysteroscopy is recently confirmed by using morcellation techniques to avoid some complications [12]. Organizing managements for term or late IPs, Nagayama et al. in a literature review presented term IPs that were managed mostly by supracervical hysterectomy [2]. Also, in other case reports of coexistence of the morbid placenta adherence with interstitial pregnancy, it is suggested to manage this category by surgical techniques using hysterectomy as well as segmental resection or medical therapy using methotrexate [3–6]. In our case, hysterectomy was done, and histopathologic study showed placenta increta. The rarity is emerging of the placenta from the serosa to the myometrium (Fig. 2).

In a literature review, it was shown that about 30% of interstitial pregnancies suffer from fetal growth restriction [3]. Although the exact cause is unknown, it was attributed to abnormal vasculature and eccentric position of the gestational sac and in a lesser extent, fibrin material deposition [3, 6, 13]. Our case sonography showed bi-parietal diameter of 97th centile, abdominal circumference of 23rd centile, and estimated fetal weight of 2851 which is the 41st centile for 36 weeks and 4 days of gestational age. Also, the weight of the delivered baby was 2840 grams, which is in the normal range for her gestational age.

The strength of our case report is that we described a term pregnancy with no complication; however, the patient had abnormal placentation of the increta emerging inversely from the serosa to the myometrium. Also, when the patient had developed with internal bleeding, she was stabilized, and her interstitial pregnancy unwittingly continued which can arise the question whether there is a subtype of this pregnancy being managed till viability of the fetus. Also, we emphasize the possible misdiagnosis of hyper-vascularity of the abnormal placentation with cancer. We should notice the absence of images of her first surgery as the limitation of our study.

In conclusion, it is likely that some conditions of interstitial pregnancy exist leading to a full term pregnancy, especially if it is missed to be recognized in routine scans during pregnancy. Physicians should be aware of such pregnancies and are suggested to pay attention to the coexistence complications such as placenta accreta to manage them more accurately.

## Data Availability

All data generated during this study are included in this published article.

## Abbreviations

IP: Interstitial pregnancy
